# Supplementary material for: The Impact of Ca2+ on Intracellular Distribution of Hemoglobin in Human Erythrocytes
Source: Cells. 2023 Sep 15;12(18):2280. doi: 10.3390/cells12182280 (PMC10526966; doi:10.3390/cells12182280)
Supplement: Supplementary file 1 [file cells-12-02280-s001.zip › cells-2567785-supplementary.pdf]

SUPPLEMENTARY DATA

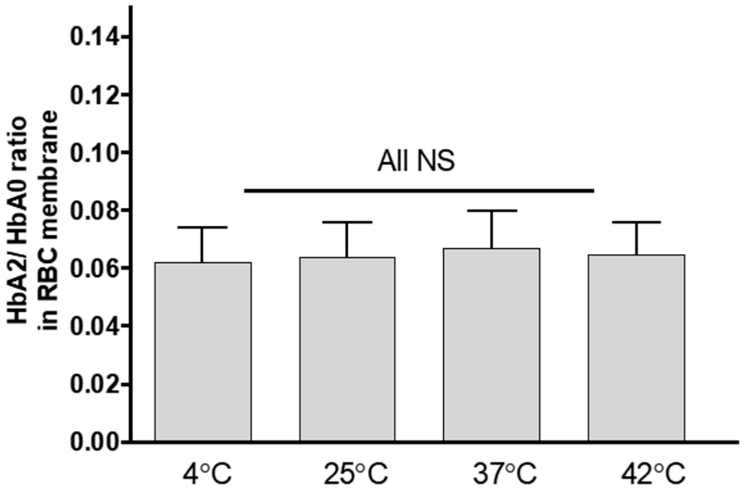

Supplementary Figure S1. Temperature-dependence of the HbA2/HbA0 ratios in pre-membrane Hb pool. EDTA-preserved whole blood was incubated at various temperatures for two hours. Then, RBCs were separated from the blood plasma and buffy coat through a quick 1700 g centrifugation. Immediately after that, the RBC membrane was isolated and the membrane distribution of Hb isoforms was measured. Both procedures are described in detail in the Materials and Methods section. Data for four independent experiments are presented as mean ± SD; No statistically significant differences (all NS, $p > 0.05$) were found for any of the incubation temperatures Student's t-test. Mean HbA2/HbA0 ratio (±SD) in intact RBCs in these experiments was 0.028 ± 0.003.

Supplementary Table S1. The numerical data related to Figures 1C and D. Percent of each Hb isoform out of total Hb is shown. Data are presented as medians ± CI (1C) and means ± SD (1D).

	N	HbF, % of total Hb		HbA2, % of total Hb		HbA0, % of total Hb	
		Intact	Membrane	Intact	Membrane	Intact	Membrane
EDTA-plasma	21	0.30±0.07	0.20±0.12	2.90±0.13	9.65±0.61	96.8±0.2	90.2±0.6
Heparin-plasma	16	0.30±0.08	0.30±0.08	2.90±0.17	4.70±0.26	96.8±0.2	94.9±0.3
Citrate-plasma	5	0.20±0.05	0.10±0.04	2.94±0.13	6.34±0.82	96.9±0.1	93.1±0.8
EDTA-plasma	6	0.37±0.2	0.33±0.30	2.82±0.15	7.03±0.9	96.82±0.21	92.6±0.9
CPDA (3 days)			0.45±0.26		7.68±0.82		91.9±0.8
CPDA (14 days)			0.20±0.15		8.30±1.28		91.5±1.2
CPDA (28 days)		0.54±0.13	0.38±0.17	2.30±0.10	7.63±0.74	97.16±0.05	92.0±0.7

Supplementary Table S2. The numerical data related to Figure 2A. Percent of each Hb isoform out of total Hb is shown. Data are presented as means ± SD.

	N	HbF, % of total Hb		HbA2, % of total Hb		HbA0, % of total Hb	
		Intact	Membrane	Intact	Membrane	Intact	Membrane
EDTA-plasma	3	0.27±0.06	0.13±0.23	3.10±0.10	8.60±1.39	96.6±0.2	91.3±1.3
PBS+2 mM Ca^{2+}							
0.25h			0.53±0.29		5.40±0.40		94.1±0.2
0.5h			0.23±0.29		5.37±0.64		94.4±0.7
1h			0.27±0.06		4.87±0.40		94.9±0.5
2h			0.30±0.10		4.00±0.10		95.7±0.2
4h			0.23±0.06		3.97±0.06		95.8±0.1
6h			0.17±0.06		4.97±0.25		94.9±0.3
24h			0.13±0.06		5.10±0.53		94.8±0.6
PMB+2 mM Ca^{2+}							
0.25h			0.10±0.10		5.13±0.40		94.8±0.5
0.5h			0.27±0.06		4.93±0.60		94.8±0.6
1h			0.27±0.06		4.47±0.40		95.3±0.5
2h			0.27±0.06		3.73±0.21		96.0±0.3
4h			0.27±0.06		3.83±0.06		95.9±0.1
6h			0.20±0.10		5.30±0.30		94.5±0.4
24h			0.17±0.15		5.20±0.61		94.6±0.5

Supplementary Table S3. The numerical data related to Figures 2B and 2C. Percent of each Hb isoform out of total Hb and Hb concentrations in intact RBC and RBC membrane are shown. Data are presented as means ± SD.

	N	HbF, % of total Hb		HbA2, % of total Hb		HbA0, % of total Hb		N	Hb concentration, mM	
		Intact	Membrane	Intact	Membrane	Intact	Membrane		Intact	Membrane
EDTA-plasma	6	0.26±0.18	0.32±0.26	2.73±0.31	8.30±1.1	97.0±0.4	91.4±1.3	6	19.40±0.76	1.39±0.10 (7.2±0.3%)
PMB+2mM Ca			0.33±0.20		4.42±0.5		95.3±0.7			2.29±0.31 (11.8±1.4%)
DPBS			0.33±0.15		4.48±0.5		95.2±0.7			2.13±0.40 (11.0±2.1%)

Supplementary Table S4. The numerical data related to Figure 3. Percent of each Hb isoform out of total Hb is shown. Data are presented as means ± SD.

	N	HbF, % of total Hb		HbA2, % of total Hb		HbA0, % of total Hb	
		Intact	Membrane	Intact	Membrane	Intact	Membrane
EDTA-plasma	3	0.20±0.00	0.03±0.06	2.87±0.21	9.27±0.93	96.9±0.2	90.7±1.0
PBS			0.07±0.06		9.17±0.90		90.8±0.9
PBS+0.1 mM Fe^{3+}			0.03±0.06		8.90±1.08		91.1±1.1
PBS+0.02 mM Zn^{2+}			0.03±0.06		9.13±1.60		90.8±1.7
PBS+2 mM Ca^{2+}			0.10±0.10		5.30±0.70		94.6±0.8
PBS+1 mM Cu^{2+}			0.07±0.06		8.33±1.31		91.6±1.4
PBS+1 mM Mg^{2+}			0.07±0.06		8.73±1.17		91.2±1.2

Supplementary Table S5. The numerical data related to Figure 4. Percent of each Hb isoform out of total Hb is shown. Data are presented as means ± SD.

	N	HbF, % of total Hb		HbA2, % of total Hb		HbA0, % of total Hb	
		Intact	Membrane	Intact	Membrane	Intact	Membrane
PBS	6	0.40±0.00	0.20±0.26	2.77±0.29	7.58±1.66	97.1±0.1	91.0±1.2
+0.125 mM Ca^{2+}			0.13±0.15		6.53±1.82		91.8±0.6
+0.25 mM Ca^{2+}			0.13±0.15		5.60±1.20		93.3±0.1
+0.5 mM Ca^{2+}			0.20±0.10		5.22±1.05		93.8±0.7
+1 mM Ca^{2+}			0.20±0.10		5.00±0.90		94.2±1.0
+2 mM Ca^{2+}			0.30±0.10		3.80±0.43		95.6±0.2
PMB	4	0.35±0.17	0.38±0.10	3.05±0.26	7.15±0.52	96.6±0.4	92.5±0.5
+0.125 mM Ca^{2+}			0.38±0.10		5.68±0.56		94.0±0.6
+0.25 mM Ca^{2+}			0.38±0.10		5.65±0.66		94.0±0.7
+0.5 mM Ca^{2+}			0.40±0.08		5.28±0.37		94.3±0.3
+1 mM Ca^{2+}			0.40±0.08		5.15±0.65		94.5±0.7
+2 mM Ca^{2+}			0.40±0.08		4.95±0.47		94.7±0.5

Supplementary Table S6. The numerical data related to Figure 5. Percent of each Hb isoform out of total Hb is shown. Data are presented as medians ± CI.

	N	HbF, % of total Hb		HbA2, % of total Hb		HbA0, % of total Hb		N	Hb concentration, mM	
		Intact	Membrane	Intact	Membrane	Intact	Membrane		Intact	Membrane
Heparin-Plasma	6	0.20±0.56	0.30±0.75	2.85±0.28	5.40±0.77	97±0.7	94.1±1.3	7	20.7±1.06	1.13±0.19 (5.4±1.0%)
Heparin-Plasma+ 5 mM EDTA			0.15±0.90		6.90±1.05		93.0±1.6			0.73±0.22 (3.5±1.1%)
Heparin-Plasma+ 5 mM EDTA-> Heparin-Plasma			0.15±0.76		5.10±0.51		94.7±1.0			1.48±0.38 (7.4±1.7%)
PBS+2mM Ca^{2+}			0.10±0.71		4.70±0.78		95.2±1.3			1.40±0.26 (6.8±1.3%)
PBS+2mM Ca^{2+}+ 5 mM EDTA			0.15±0.75		5.55±0.75		94.2±1.1			1.28±0.37 (5.9±1.9%)
PBS+2mM Ca^{2+}+ 5 mM EDTA-> PBS+2mM Ca^{2+}			0.20±0.56		3.50±0.49		96.2±0.6			1.83±0.62 (9.1±2.8%)
PMB +2mM Ca^{2+}			0.15±0.76		5.15±0.85		94.6±1.4			1.17±0.13 (5.7±0.5%)
PMB +2mM Ca^{2+}+ 5 mM EDTA			0.10±0.83		5.70±0.81		94.2±1.3			0.73±0.17 (3.3±0.9%)
PMB +2mM Ca^{2+}+ 5 mM EDTA-> PMB +2mM Ca^{2+}			0.20±0.63		3.70±0.40		96.0±0.8			1.98±0.58 (9.3±2.8%)

Supplementary Table S7. The numerical data related to Figure 7. Percent of each Hb isoform out of total Hb is shown. Data are presented as means ± SD.

	N	HbF, % of total Hb		HbA2, % of total Hb		HbA0, % of total Hb	
		Intact	Membrane	Intact	Membrane	Intact	Membrane
PBS	9	0.28±0.10	0.21±0.13	3.02±0.18	5.63±0.92	96.7±0.3	94.2±0.8
PBS+10 μM A23187			0.27±0.11		6.08±1.50		93.7±1.5
PBS+0.125 mM Ca^{2+}			0.31±0.17		4.84±0.69		94.8±0.6
PBS+ 0.125mM Ca+ 10 μM A23187			0.28±0.13		7.63±1.65		92.1±1.7
PBS +0.5 mM Ca^{2+}			0.27±0.12		4.57±0.48		95.2±0.5
PBS+ 0.5mM Ca+10 μM A23187			0.29±0.12		7.13±2.00		92.6±2.1
PBS+2 mM Ca^{2+}			0.24±0.09		4.10±0.59		95.7±0.6
PBS+2mM Ca+10 μM A23187			0.28±0.08		4.74±1.27		95.0±1.3

Supplementary Table S8. The numerical data related to Figure 8B. Percent of each Hb isoform out of total Hb is shown. Data are presented as means ± SD.

	N	HbF, % of total Hb		HbA2, % of total Hb		HbA0, % of total Hb	
		Intact	Membrane	Intact	Membrane	Intact	Membrane
NaCl 150 mM	3	0.23±0.15	0.33±0.15	3.03±0.06	3.80±0.87	96.7±0.2	95.9±0.9
NaCl 100 mM/ KCl 50 mM			0.23+0.15		4.20+0.17		95.6+0.3
NaCl 50 mM/ KCl 100 mM			0.23+0.15		4.43+0.42		95.3+0.6
KCl 150 mM			0.23+0.15		4.27+0.31		95.5+0.4

Supplementary Table S9. The numerical data related to Figure 9. Percent of each Hb isoform out of total Hb is shown. Data are presented as medians ± CI range).

	N	HbF, % of total Hb		HbA2, % of total Hb		HbA0, % of total Hb	
		Intact	Membrane	Intact	Membrane	Intact	Membrane
PMB+2mM Ca	8	0.25±0.25	0.25±0.29	2.80±0.13	4.30±0.48	96.9±0.23	95.2±0.56
PMB+2mM Ca+ 5 mM EDTA			0.65±0.27		6.75±0.6		92.8±0.65
PMB+2mM Ca+ 50 μM DIDS			0.45±0.32		4.60±0.5		95.2±0.62
PMB+2mM Ca+ 2 mM $ZnCl_2$			0.65±0.10		7.40±0.62		91.9±0.69
